# Molecular tools for decoding multicellular systems: from mechanisms to medicine

**DOI:** 10.1093/nsr/nwaf473

**Published:** 2025-11-06

**Authors:** Jie P Li, Weiming Guo, Peng Zou, Coco Chu, Jiarui Wu, Zijian Guo, Yan Huang, Junlin Yang, Peng R Chen

**Affiliations:** State Key Laboratory of Coordination Chemistry, School of Chemistry and Chemical Engineering, Chemistry and Biomedicine Innovation Center (ChemBIC), Nanjing University, Nanjing 210023, China; Key Laboratory of Bioorganic Chemistry and Molecular Engineering of Ministry of Education, Peking University, Beijing 100871, China; Key Laboratory of Bioorganic Chemistry and Molecular Engineering of Ministry of Education, Peking University, Beijing 100871, China; Synthetic and Functional Biomolecules Center, Beijing National Laboratory for Molecular Sciences, College of Chemistry and Molecular Engineering, Peking University, Beijing 100871, China; Key Laboratory of Bioorganic Chemistry and Molecular Engineering of Ministry of Education, Peking University, Beijing 100871, China; Synthetic and Functional Biomolecules Center, Beijing National Laboratory for Molecular Sciences, College of Chemistry and Molecular Engineering, Peking University, Beijing 100871, China; Institute for Immunology, School of Basic Medical Sciences, Beijing Key Laboratory for Immunological Research on Chronic Diseases, Tsinghua-Peking Center for Life Sciences, Tsinghua University, Beijing 100084, China; Key Laboratory of Systems Biology, CAS Center for Excellence in Molecular Cell Science, Shanghai Institute of Biochemistry and Cell Biology, Chinese Academy of Sciences, University of Chinese Academy of Sciences, Shanghai 200031, China; State Key Laboratory of Coordination Chemistry, School of Chemistry and Chemical Engineering, Chemistry and Biomedicine Innovation Center (ChemBIC), Nanjing University, Nanjing 210023, China; Department of Chemical Sciences, National Natural Science Foundation of China, Beijing 100085, China; Department of Chemical Sciences, National Natural Science Foundation of China, Beijing 100085, China; Key Laboratory of Bioorganic Chemistry and Molecular Engineering of Ministry of Education, Peking University, Beijing 100871, China; Synthetic and Functional Biomolecules Center, Beijing National Laboratory for Molecular Sciences, College of Chemistry and Molecular Engineering, Peking University, Beijing 100871, China

**Keywords:** cell-cell communication, molecular tools, proximity labeling, spatial omics, precision medicine

## Abstract

Cell-cell communication (CCC) is fundamental to essential biological processes including growth, differentiation, immune surveillance, and tissue homeostasis, and its dysregulation underlies various diseases such as cancer, autoimmunity, and neurodegeneration. In response to growing interest in decoding complex multicellular interactions, the 380th Shuangqing Forum entitled ‘Chemical, Biological, and Medical Frontiers in Multicellular Complex Systems’ was convened, providing a platform to discuss recent interdisciplinary breakthroughs. This review, emerging from forum discussions, highlights the latest advancements in molecular tools—such as super-resolution imaging, proximity labeling, bioorthogonal chemistry, synthetic receptors, and single-cell spatial omics—that enable unprecedented insights into spatial, molecular, and functional aspects of CCC. Emphasizing their translational potential, we discuss their profound implications for immuno-oncology, regenerative medicine, and autoimmune diseases. We further outline current challenges and opportunities, particularly advocating for a future precision medicine framework centered around targeted modulation of cell-cell interactions.

## Teaser

This review highlights emerging molecular tools for decoding cell-cell communication and discusses their translational potential in addressing clinical challenges, ultimately paving the way toward a precision-medicine paradigm centered on the targeted perturbation of multicellular systems.

## INTRODUCTION

**Figure 1.**
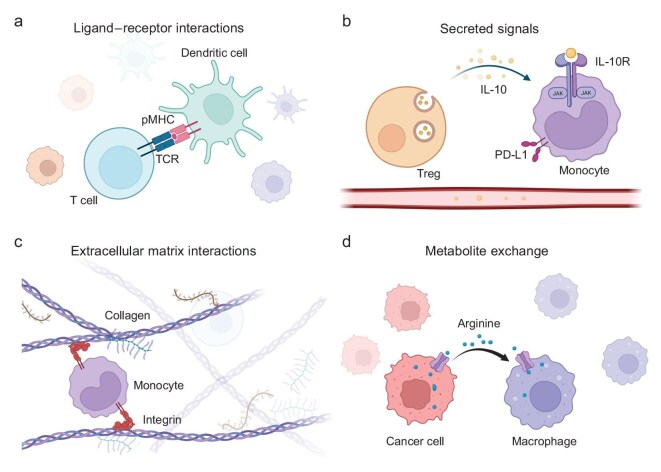
Different types of cell-cell interactions and communication. (a) Ligand–receptor interactions: ligands on sender cells binding to receptors on receiver cells, triggering intracellular signaling cascades. In adaptive immunity, dendritic cells present antigens to T cells via pMHC, activating T cell receptors (TCRs) and co-stimulation for immune responses [14]. (b) Secreted signals: cell-cell interactions mediated by diffusible signaling molecules released by sender cells and detected by receiver cells. In colorectal cancer-derived liver metastases, Treg-derived IL-10 upregulates PD-L1 expression in monocytes and reduces antitumor immunity [15]. (c) Extracellular matrix interactions: bidirectional communication between cells and their surrounding extracellular matrix. In inflammatory response, integrin mediates monocyte adhesion and transmigration by binding with collagen, directing selective recruitment of cells to the interstitial sites of inflammation [16]. (d) Metabolite exchange: the transfer of metabolic intermediates between cells, tissues, and their microenvironment, facilitating metabolic coordination and homeostasis. In the tumor microenvironment, breast cancer cells serve as the primary source of arginine, which induces a pro-tumor polarization of tumor-associated macrophages [17].

Cell-cell communication (CCC), the essential process by which cells exchange signals, governs cell behavior throughout life, from embryogenesis to adulthood. CCC orchestrates critical processes such as growth, differentiation, immune surveillance, tissue repair, and homeostasis [1]. Cells utilize diverse communication modes, ranging from local physical contacts between paracrine cell pairs to secreted endocrine signals that act on distant effector cells (Fig. 1). Mechanistically, CCC commonly involves sender cells producing ligand molecules that bind to specific receptors on the receiver cells, triggering intracellular signal cascades (Fig. 1a) [2]. Beyond classical ligand-receptor interactions, cells also communicate through membrane-bound signals, direct cytoplasmic connections, extracellular matrix interactions, secreted signals, and exchange of metabolites (Fig. 1b–d).

Early studies highlighted the importance of CCC by demonstrating that dissociated sponge cells could reassemble into functional groups [3], and amphibian embryonic cells selectively reorganize to form structured tissues [4]. These observations established cellular communication and adhesion as fundamental to multicellular organization. Subsequently, core signaling pathways such as Notch [5], Wnt [6], Hedgehog [7], and TGF-β [8] were identified as evolutionarily conserved mediators of intercellular interactions across diverse biological contexts. In modern biology, effective CCC underpins normal physiology, exemplified by immune T cells engaging antigen-presenting cells for adaptive immunity, neuronal synapses transmitting electrochemical signals, and epithelial cells forming tight junctions for barrier maintenance. Disruptions or miscommunications within CCC pathways result in severe consequences, including developmental abnormalities, cancer progression, degenerative diseases, and metabolic disorders. Decoding these intricate communication networks remains vital for elucidating how coordinated cellular interactions sustain health or lead to disease.

Traditionally, CCC has been investigated through bulk biochemical assays and genetic perturbations to identify core signaling pathways, such as the Notch, cytokine, and growth factor pathways. Classical protein-protein interaction methodologies, including immunoprecipitation, yeast two-hybrid screening, and Förster resonance energy transfer (FRET) imaging, have provided valuable insights into specific molecular interactions [9]. However, these methods often focus on isolated protein partners and/or homogenized cell populations, missing the broader context of multicellular organization. In reality, each cell’s phenotype is shaped by myriad simultaneous inputs from neighboring cells and the extracellular milieu. A single cell type can engage in distinct crosstalk with different partners, and signaling outcomes depend on spatial arrangement, timing, and the collective state of the cellular community [1]. Capturing this ‘social network’ of cells in their native context within the tissue—who interacts with whom, where, when, and how—poses a formidable challenge that has driven the development of innovative technologies.

Over the past decade, a suite of molecular tools has emerged to visualize and map cell-cell interactions with unprecedented resolution and scale. These approaches integrate advances in chemistry, biology, and engineering to move beyond simple pairwise assays and observe cell communication as it occurs in complex systems. For instance, new imaging modalities allow direct visualization of interacting cells in living organisms [10], while bioorthogonal chemical probes can tag and capture the molecular exchanges at cell contact sites [11]. Likewise, single-cell and spatial ‘-omics’ methods can profile the signaling molecules exchanged between neighboring cells across entire tissues [12]. Cell engineering strategies even enable us to induce or record specific cell-cell interactions via synthetic receptors and genetic circuits [13]. Collectively, these tools are illuminating the mechanisms of intercellular dialogue in rich detail, identifying the key molecular players and interaction topologies that govern multicellular function.

In this review, we survey the spectrum of molecular technologies for decoding multicellular systems, and discuss how they are advancing both fundamental biology and translational medicine. We begin by examining current methods to monitor and manipulate cell-cell interactions, from high-resolution imaging to chemical tagging and engineered cell-cell sensors. We then highlight translational applications, illustrating how insights from these tools are informing new therapies. Next, we consider the challenges and emerging opportunities in the field, including technical hurdles in resolving complex cell networks and prospects for integrating multi-modal data. Finally, we outline a forward-looking framework for precision medicine that leverages intercellular communication maps to guide targeted interventions. By bridging molecular mechanisms to medical applications, we aim to show how decoding multicellular communication can pave the way for innovative strategies of personalized medicine. This convergence of exploratory tools and clinical insight is ushering in an era in which the cellular ‘dialogues’ underlying physiology and disease can be understood and even modulated with remarkable precision.

## MOLECULAR TOOLS FOR DECODING MULTICELLULAR SYSTEMS

Elucidating cell interactions within tissues necessitates tools capable of capturing spatial, molecular, and functional complexities simultaneously. Currently, a diverse array of molecular technologies enables visualization of interacting cells, identification of exchanged signals, and perturbation or recording of these interactions in living systems. These methodologies encompass imaging techniques that directly observe cellular contacts, chemical proximity labeling approaches, as well as bioorthogonal chemical methods that highlight molecular features at cell interfaces, synthetic biology strategies leveraging engineered receptors or circuits to detect and modulate interactions, and single-cell or spatial omics methods that reconstruct communication networks from molecular data. Each class of method provides a unique window into multicellular organization, and together they are beginning to give an integrated view of intercellular communication.

### Visualization of cell-cell interactions: advanced imaging techniques

**Figure 2.**
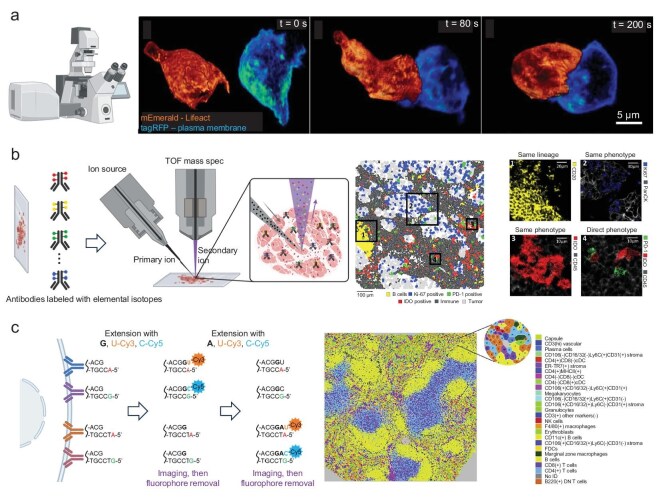
Advanced imaging techniques for direct visualization of cell-cell communications. (a) Observation of 4D cell-cell interactions via super-resolution fluorescence microscopy [24]. Copyright © 2014, American Association for the Advancement of Science. (b) Mass spectrometry-based imaging techniques by using metal isotope tagged antibodies to visualize multiplex protein targets simultaneously in the tissue sample [27]. Copyright © 2018, Elsevier Inc. (c) Multiplexed DNA-tagged antibody staining in CODEX to analyze multiple immune cell types and their interactions [28]. Copyright © 2018, Goltsev *et al.* Published by Elsevier Inc.

One of the most intuitive ways to decode multicellular systems is to directly visualize cells interacting within their native contexts (Fig. 2). Microscopy has long been essential in cell biology, and recent advances have significantly enhanced imaging capabilities, allowing observation of cell-cell interactions with unprecedented depth, spatial resolution, and multiplexed molecular detail. Conventional fluorescence microscopy uses distinct fluorescent labels to identify adjacent cells or cell-cell contacts in cultured systems or tissue sections. Advances in two-photon intravital microscopy have particularly improved live imaging deep within intact tissues, enabling immunologists to dynamically track cells *in vivo*, such as T cells scanning lymph nodes and forming transient synapses with antigen-presenting dendritic cells [10,18]. Such time-lapse intravital approaches first revealed critical dynamic behaviors, including the ‘kiss-and-run’ interactions of immune cells, and remain invaluable for real-time analysis of cellular contacts and migrations. *Ex vivo* confocal and multiphoton microscopy, applied to organ explants or engineered 3D co-cultures, further enable detailed visualization of spatial arrangements in embryonic organoids or tumor immune infiltrates [19]. Additionally, recent technical innovations have significantly expanded the multiplexing capabilities of two-photon intravital microscopy. Modern setups incorporate broad-spectrum pulsed lasers, spectral detectors, and computational spectral unmixing to simultaneously image up to five fluorescent channels alongside second-harmonic generation signals. Innovative single-wavelength three-photon excitation approaches (e.g. 1340 nm pulses) further facilitate concurrent excitation of multiple fluorophores, dramatically boosting signal intensity for deep-tissue multicolor imaging [20]. The development of bright near-infrared fluorescent proteins reduces spectral overlap and extends imaging depth, greatly enhancing the clarity and resolution of complex cellular interactions in living organisms [21].

To push the limits of spatial resolution at cell junctions, super-resolution fluorescence microscopy (such as photoactivated localization microscopy (PALM) [22], stimulated emission depletion (STED) microscopy [23], or lattice light-sheet microscopy [24]) has been applied to visualize the nano-scale organization of cell-cell contact sites. These methods can resolve microdomains within synapses or junctions, revealing patterns such as the segregation of T cell receptor microclusters and adhesion molecules within the immunological synapse. Similarly, live-cell lattice light-sheet imaging has allowed researchers to follow cell interactions in 4D (three dimensions over time) with minimal phototoxicity, capturing, for instance, the sequential contacts among cells in a developing embryo or neural progenitors migrating and touching in brain organoids (Fig. 2a) [24]. On the other end of the scale, whole-organ tissue clearing and light-sheet imaging techniques now enable us to visualize cellular interaction networks across large volumes—for example, mapping how blood vessels and nerves intermingle throughout an organ [25], or how disseminated tumor cells interact with the distant microenvironment in metastasis [26]. These imaging advances paint a vivid picture of the architecture of cell-cell interactions, from nanometer-scale molecular assemblies to centimeter-scale tissue-wide cell networks.

Multiplexed imaging of molecular markers is another powerful approach to decode multicellular systems. Traditional immunofluorescence is generally limited to a handful of colored labels at once, but new techniques overcome this limitation to profile dozens of proteins or RNAs *in situ* while preserving spatial relationships. Methods such as imaging mass cytometry and multiplexed ion beam imaging (MIBI) use metal-tagged antibodies detected by mass spectrometry to visualize 30–40 proteins simultaneously in a tissue section (Fig. 2b) [27]. Likewise, DNA-barcode labeling and iterative staining in technologies like CODEX (Co-Detection by Indexing) can achieve highly multiplexed immunostaining (>50 markers) on cells in tissue (Fig. 2c) [28]. These approaches allow the identification of many cell types and their functional states all at once, and critically, reveal how they are arranged relative to each other. For instance, in a tumor biopsy one can simultaneously visualize diverse immune cell subsets, stromal cells, and cancer cells and determine which cell types are in direct contact or organized into neighborhoods [27]. Characteristic spatial patterns, such as T cells clustering at the invasive tumor margin in association with macrophages, can indicate defined cell-cell interaction networks, exemplified by T cells engaging macrophages that potentially present tumor antigens [29]. By integrating multiplexed imaging with advanced image analysis and machine learning algorithms, researchers are now systematically mapping cellular communities and interaction niches within tissues through unbiased, data-driven approaches. These spatially resolved cell atlases are a rich resource for discovering new interactions and understanding how the configuration of cells in a microenvironment influences function.

In summary, advanced imaging offers a direct visual approach to decode multicellular systems, from dynamic live-cell contacts to static molecular snapshots of tissue architecture. A limitation, however, is that seeing cells together does not always reveal the biochemical nature or consequence of their interaction. Thus, imaging is increasingly complemented by molecular tagging methods that can report on the biochemical exchanges and physical proximities at cell-cell interfaces.

### Proximity labeling and bioorthogonal chemistry: tagging the cell-cell interface

**Figure 3.**
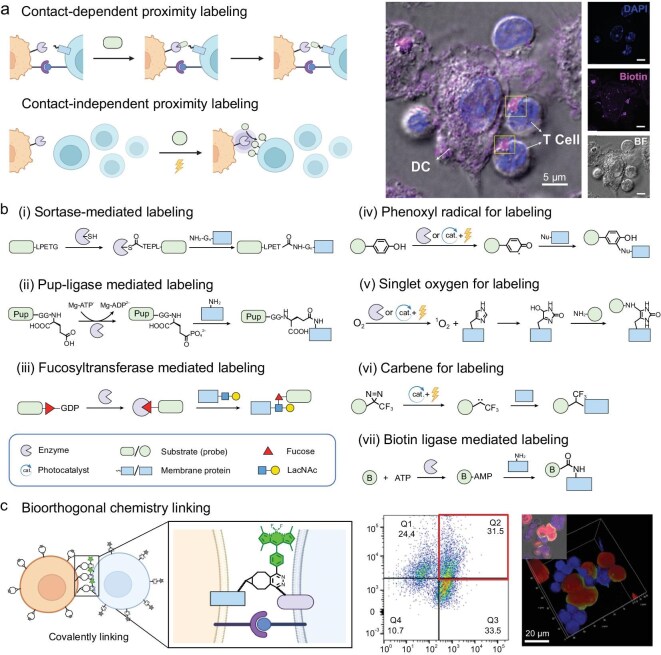
Methodologies for proximity labeling and bioorthogonal ‘turn-on’ reactions to profile cell-cell communication interfaces. (a) The scheme and example of contact-dependent and contact-independent proximity labeling [34]. Copyright © 2022, American Chemical Society. (b) The detailed chemistry in intercellular proximity labeling: (i) SrtA transfers its substrate, LPETG peptide, to proximal N-term glycine-rich proteins. (ii) The Pup ligase phosphorylates the Pup substrate which covalently tags the lysine residues on proximal proteins. (iii) The fucosyltransferase labels GDP-fucose on the LacNAc on the cell surface. (iv) Peroxidases or photocatalysts generate reactive tags from phenol for labeling its surrounding proteins. (v) Photocatalytic proteins or photocatalysts generate singlet oxygen for proximity labeling. (vi) Photocatalysts generate reactive carbenes from a diazirine to label the proximal proteins. (vii) Biotin ligases catalyze the formation of a reactive biotin-AMP tagging the lysine residues on neighboring proteins. (c) Bioorthogonal ‘turn-on’ reactions illuminating the interface of cell-cell interactions [37]. Copyright © 2024, Xue *et al.* Published by American Chemical Society.

While microscopy reveals which cells are in a neighborhood, proximity labeling techniques delve deeper—illuminating the molecular exchanges that occur at the interface of two physically interacting cells (Fig. 3a). These methods harness either enzymatic activity or bioorthogonal chemical reactions to selectively tag proteins and other biomolecules that reside within, or transit across, the cell-cell contact zone. By capturing molecular information precisely at the moment of contact, proximity labeling offers a snapshot of the biochemical dialogue between cells, complementing spatial imaging with interaction-specific molecular readouts.

A seminal example of this approach is LIPSTIC (Labeling Immune Partnerships by SorTagging Intercellular Contacts) [30], which employs the bacterial transpeptidase Sortase A to covalently transfer a biotin-tagged peptide from a donor to an acceptor cell during receptor–ligand-mediated interactions (Fig. 3b). Initially, the system required engineered expression of defined receptor pairs (e.g. CD40L-CD40) to bring the enzyme and substrate into proximity at immune synapses. To expand its versatility, a generalized version—universal LIPSTIC (uLIPSTIC) [31]—was later developed. By non-specifically anchoring Sortase A and its peptide substrate to opposing cell membranes, uLIPSTIC enables labeling of any physical contact occurring within 14 nm, independent of specific molecular recognition. This contact-dependent enzymatic reaction effectively ‘barcodes’ interacting cell pairs, preserving a covalent record of engagement for subsequent recovery and transcriptomic analysis. In mouse models, LIPSTIC has enabled direct quantification and isolation of dendritic cells that formed immune synapses with CD4⁺ T cells, as well as mapping broader cellular interaction networks across immune and non-immune cell types. When combined with single-cell RNA sequencing, the method facilitates construction of *in vivo* ‘interaction atlases,’ linking physical contact to downstream transcriptional responses. Unlike computational inference methods, this strategy directly captures contact events in complex tissues, providing an unbiased and temporally resolved molecular readout of cellular communication.

Beyond Sortase-based systems, several orthogonal chemical strategies have emerged to label cellular interactions with varying specificity, modularity, and *in vivo* compatibility. FucoID [32] employs a bacterial fucosyltransferase to transfer biotinylated fucose analogs from engineered donor cells onto *N*-acetyl-D-lactosamine (LacNAc) structures on juxtaposed cell surfaces (Fig. 3b), thus capturing endogenous contacts without genetic modification of the recipient cell. This has proven particularly effective in isolating antigen-specific T cells from tumor or vaccine responses. EXCELL (Enzyme-mediated EXtracellular Cell Labeling) [33] further improves generalizability by using engineered Sortase mutants that recognize a broad range of unmodified N-terminal glycine residues on neighboring cells, enabling covalent tagging without a defined receptor-ligand pair or pre-installed acceptor motifs. More recently, photocatalytic proximity labeling strategies—such as PhoXCELL (photocatalytic proximity cell labeling) [34] or ruthenium-based systems [35]—have introduced temporal precision by enabling contact-dependent labeling upon light activation (Fig. 3b) [36]. These systems typically anchor a photocatalyst (e.g. Ru(bpy)_3_^2+^) to the surface of donor cells, where light-induced reactive species mediate covalent labeling of adjacent membranes. DNA-directed versions further increase spatial specificity by localizing the photocatalyst via hybridization only when cells are in direct contact. These systems offer rapid and tunable labeling kinetics but remain limited by issues of light penetration and phototoxicity in deep tissue applications.

Each platform presents trade-offs in terms of engineering requirements, labeling resolution, and compatibility with *in vivo* systems. Enzymatic methods like LIPSTIC and EXCELL offer stable covalent tags with minimal off-target effects, but typically require genetic engineering of at least one cell population. In contrast, FucoID and photocatalytic systems enable broader applicability to primary cells or clinical samples, with FucoID excelling in surface glycan labeling and light-triggered platforms providing precise temporal control. Together, these tools form a growing chemical toolkit to map dynamic intercellular networks, each capturing distinct dimensions of the cell-cell interactome.

Beyond proximity labeling chemistry, bioorthogonal chemical reactions have become powerful tools to probe cell-cell interfaces. Bioorthogonal chemistry—reactions that can occur inside living systems without perturbing native biochemistry—allows scientists to introduce artificial tags or even forge covalent bonds between cells in a highly specific manner. A striking recent example used a two-component chemical system to both detect and induce cell-cell interactions via click chemistry [37]. In this strategy, immune cells such as macrophages were decorated with a maleimide-appended tetrazine-caged boron dipyrromethene (BODIPY)-based fluorophore, and cancer cells were modified with a maleimide-substituted bicyclo[6.1.0]non-4-yne (BCN) on its surface. When the two cell types came into contact, the tetrazine and BCN underwent an inverse electron-demand Diels–Alder reaction, a classic click reaction, that covalently linked the cells together (Fig. 3c). This bioorthogonal coupling had two effects: (1) because the reaction cleaved the quenching tetrazine caging group, it restored fluorescence of the dye at the interface, illuminating the areas where cells came into contact with each other; and (2) it literally ‘stapled’ the cells together, strengthening their interaction. In the reported case, macrophages bound to cancer cells via this chemical tether exhibited enhanced phagocytosis of the tumor cells. Thus, the method not only visualized the cell-cell contact through a fluorescent signal but also functionally augmented it, highlighting the potential of chemical biology to manipulate cell interactions. The utilization of bioorthogonal chemistry, which operates without perturbing native cellular processes, allows for its application *in vivo*. It facilitates the promotion of targeted cell-cell interactions, such as enhancing immune cell engagement with tumor cells, while concurrently labeling these interactions for subsequent analysis.

Another set of chemical tools focuses on tagging the molecular exchange between cells. Cells constantly secrete and absorb metabolites, proteins, and other factors; tracking these intercellular transfers can illuminate functional interactions. Metabolic labeling with bioorthogonal tags is one technique used to follow such exchanges. For example, cells can be fed with metabolic precursors bearing unique chemical handles, such as azide or alkyne groups, that are subsequently incorporated into secreted metabolites or surface-expressed glycans [38]. Any neighboring cell that takes up or reacts with these labeled molecules can then be identified by adding a fluorescent or affinity probe that clicks to the handle. Pioneering work used azide-labeled sugars to tag glycoproteins on cell surfaces [39]; this has been further extended to visualize glycan-mediated cell-cell interactions, such as the binding of immune cells to sialylated glycans on cancer cells, by fluorescently detecting the localization of azide-tagged glycans. Similarly, a cell’s secretome can be metabolically labeled using heavy isotopes or clickable amino acids, allowing for the subsequent tracing into recipient cells through proteomic analysis. These approaches help map functional connectivity, for instance, identifying which cell types are supplying key metabolites that others depend on, or which cells uptake a drug metabolite produced by a different cell type.

In summary, proximity labeling and bioorthogonal chemistry provide a versatile toolkit to decipher molecular interactions at cell-cell interfaces. Enzymatic approaches like LIPSTIC [30] create lasting molecular tags of cellular partnerships *in vivo*, enabling precise downstream analysis. Bioorthogonal chemical probes further extend this toolkit by detecting, manipulating, and functionally modulating intercellular interactions. Looking forward, genetically encoded photo-crosslinking probes, such as benzophenone or diazirine-modified amino acids, might systematically uncover novel transient receptor-ligand pairs, capturing fleeting interactions through covalent linkage upon UV activation [40]. Proximity ligation assays (PLAs) could subsequently validate these newly identified interactions, providing confirmatory evidence of molecular proximities [41]. Collectively, these complementary chemical methods move beyond simple visualization to comprehensively decode intercellular communication at the molecular level.

### Engineered receptors and genetic circuits: sensing and programming cell interactions

**Figure 4.**
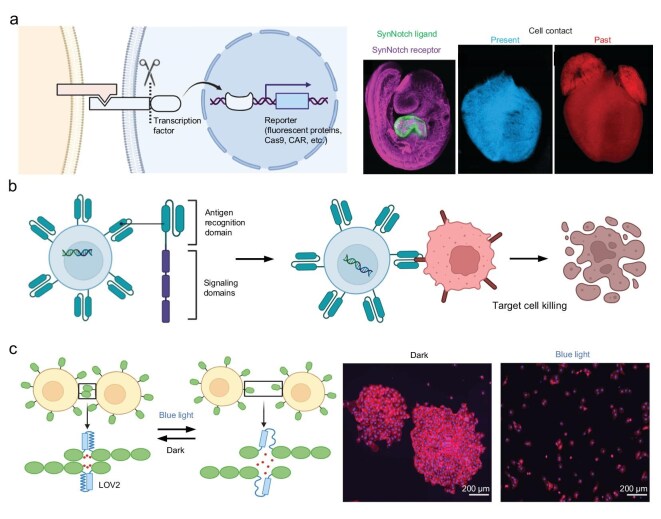
Engineered receptors and genetic circuits to program cell-cell interaction. (a) SynNotch system. Cell-cell contacts can be monitored and recorded by replacing the ligand-binding domain and the transcriptional domain of native Notch with the designed parts [47]. Copyright © 2022, The American Association for the Advancement of Science. (b) CAR-T cells can kill target cells with the CAR-recognized antigens. (c) Optogenetic tools for controlling cell-cell adhesion [51]. Copyright © 2023, Mombo *et al.* Published by Springer Nature.

Chemical and imaging tools passively observe or tag existing cell interactions, but synthetic biology approaches allow us to actively engineer cells to report or modulate their interactions. By rewiring cellular signaling pathways, one can create living sensors that detect when a cell contacts a specific neighbor and then execute a programmed response, effectively translating a cell-cell interaction into a measurable or useful output. Such tools are helping researchers dissect complex interaction networks and also hold promise for therapeutic applications where cells can be programmed to carry out defined actions in multicellular environments (Fig. 4).

A landmark development in this area is the design of synthetic Notch (synNotch) receptors [13]. Notch is a native cell-contact signaling pathway in which a membrane-tethered transcription factor is released when the receptor’s external domain binds a ligand on a neighboring cell. The synNotch strategy preserves this basic mechanism, an inducible proteolysis upon cell contact, but replaces both the ligand-binding domain and the intracellular transcriptional domain with user-specified parts. The result is a highly customizable receptor that can be engineered to recognize virtually any surface protein as the trigger and drive any chosen gene expression program as the output (Fig. 4a). For example, one can create a synNotch receptor that recognizes a tumor antigen on another cell and, when engaged, causes the T cell bearing it to express a fluorescent protein or secrete a specific cytokine [42]. SynNotch receptors thus act as sentinels that convert a cell-cell interaction into a gene expression change. Because the synNotch signaling cascade is synthetic and insulated from the cell’s native pathways, multiple different synNotch receptors can be introduced into the same cell without crosstalk. This enables the implementation of logic-gated cellular responses. For instance, a cell can be engineered to require simultaneous contact with two distinct cell types—each recognized through a different synNotch—before initiating a functional response [43]. Alternatively, the system can be designed to perform sequential actions, whereby an initial cell-cell interaction induces the engineered cell to produce and secrete a ligand that subsequently modulates the behavior of a third cell type, effectively creating a controlled signaling cascade [44]. Using synNotch, scientists have demonstrated circuits like T cells that, upon contacting a target cell, begin secreting bespoke combinations of cytokines or engage in ‘cascading’ interactions that organize other cells [45]. SynNotch-based cell circuits have even been used to pattern multicellular arrangements, for instance, engineering cells that self-assemble into spatial patterns by having one cell type instruct its neighbors to differentiate, mirroring developmental processes but in a designed way [46]. For decoding multicellular systems, synNotch can be harnessed as a tool to report interactions: by driving a fluorescent reporter or a DNA-barcoded ‘interaction log’ only when two cells of interest meet, providing a readable record of specific cell contacts analogous to proximity tagging but via a biological mechanism [47].

In parallel, chimeric antigen receptors (CARs) [48] on T cells represent another form of engineered receptor that recognizes a target cell and triggers a defined response—in this case, activation and killing of the target cell. CAR-T cells, which carry a synthetic receptor for a tumor antigen, exemplify how engineering cell-cell recognition has revolutionized medicine (Fig. 4b). Although CARs are predominantly employed as a therapeutic modality, they also function as valuable tools for probing cellular interactions. The clinical success of CAR-T cell therapies demonstrates that T cells can be directed to engage specific cell types and modulate those interactions by eliminating the target. Newer iterations of CARs have integrated logic gates, such as AND/OR gating similar to synNotch systems, to enhance target specificity and therapeutic safety, effectively requiring dual cell-cell interactions to fully activate the T cell [42]. Such advances blur the line between decoding and reprogramming multicellular networks—cells can be re-engineered not only to understand who they are talking to but to change how they talk.

Beyond receptors, synthetic biology offers genetic circuits that permanently record cellular interactions or states into genomic DNA, creating a memory of an event. CRISPR-based recording systems can be designed to introduce mutations or genomic barcodes by activating CRISPR-related nucleases when receiving specific signals, such as the binding of ligands from adjacent cells to synthetic receptors [49]. Subsequent sequencing of these barcodes can tell us which signals each cell experienced over time. While still early in development, researchers have conceptualized using this to record cell-cell interactions. For instance, a two-cell interaction could bring together two halves of a split enzyme that then triggers a DNA edit, marking both cells’ genomes to indicate ‘we met’ [50]. Adapting such molecular recorders to mammalian cell communities could allow retrospective reading of interaction histories during development or disease progression, effectively mapping the contact lineage that cells have experienced.

Engineered circuits can also modulate interactions in more direct ways. Optogenetic tools, for instance, can control cell adhesion [51]: by fusing light-sensitive dimerization domains to adhesion molecules or receptor components, one can use light to reversibly induce or disrupt specific cell-cell contacts on demand (Fig. 4c). This has been used to create dynamic patterns of cell assembly and to test how timing of contact affects cell signaling outcomes, for example, by precisely controlling the duration of adhesion between two distinct cell types, researchers can observe how defined interaction windows affect the initiation of differentiation programs. Similarly, synthetic secretion systems can be installed so that when a cell detects one partner, it releases a factor that affects another cell, allowing designed three-way or community-level interactions.

In summary, synthetic biology provides interactive tools to sense, record, and reprogram cell-cell communication. Synthetic Notch receptors exemplify how we can turn a natural contact-dependent mechanism into a customizable sensor for any cell interaction of interest. CARs and related engineered receptors show that we can not only detect but also robustly act on cell contacts, with enormous therapeutic implications. Genetic circuits promise to log interactions over time or implement multi-cellular logic, pushing towards a future where we can treat cell communities as programmable entities. These research tools help parse the sufficiency and necessity of specific interactions by letting us artificially add or block them in a controlled way. Together with observational methods, they complete the toolkit for decoding multicellular systems by enabling experimentally controlled perturbation of the cell interactome.

### Single-cell and spatial omics: inferring and mapping communication networks

**Figure 5.**
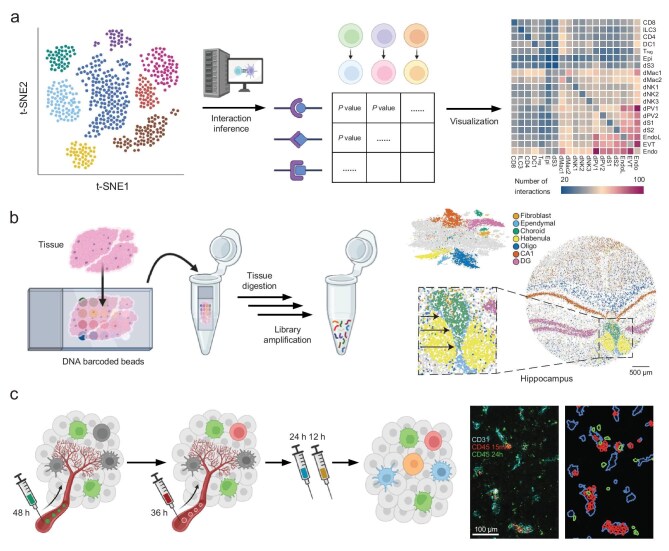
Single-cell and spatial-omics approaches to map cell-cell communication networks. (a) Computational tools to analyze scRNA-seq data for cell to reconstruct cell-cell interaction networks [52]. Copyright © 2020, The Author(s), under exclusive license to Springer Nature Limited. (b) Spatial transcriptomics techniques to capture gene expression *in situ* in tissue samples [54]. Copyright © 2019, The American Association for the Advancement of Science. (c) Zman-seq is a time-resolved scRNA-seq method to resolve cell states and molecular trajectories *in vivo* [61]. Copyright © 2023 Elsevier Inc.

The advent of single-cell ‘omics’ technologies has revolutionized our ability to chart multicellular ecosystems. By profiling genes, proteins, and metabolites at single-cell resolution, we can infer a wealth of information about cell-cell interactions, even without directly observing cells touching. Single-cell RNA sequencing (scRNA-seq), in particular, has been a driving force in decoding communication networks: from a mixed population of cells, one can identify which ligands and receptors each cell is expressing and use this to predict potential interactions between cell types [52]. While expression alone does not prove physical contact, it provides a molecular interaction map that guides further exploration (Fig. 5).

Numerous computational tools have been developed to analyze scRNA-seq data for cell communication signals. For example, databases like CellPhoneDB catalog known ligand-receptor pairs and their downstream effects [52]. When provided with single-cell transcriptomes, these tools statistically evaluate which pairs of cell types have complementary ligand-receptor expression, suggesting a putative interaction (Fig. 5a). For instance, if one cell type exhibits high expression of a cytokine gene while another displays elevated expression of the corresponding receptor, this suggests the potential paracrine signaling between the two populations. This approach was used to construct a draft ‘wiring diagram’ of human cell interactions: a network of thousands of ligand-receptor connections across human tissues has been assembled, highlighting, for instance, the central role of immune cells as communication hubs in many organs [53]. In this study, single-cell sequencing of 144 different human cell types enabled inference of which pairs are most likely to engage in signaling and through which pathways. Such analyses pointed to novel interactions, some of which were later validated experimentally, and confirmed known ones, such as the extensive crosstalk between stromal and immune cells in inflammatory niches.

A key limitation of interaction maps derived from scRNA-seq is the loss of spatial context. Cells that appear to ‘interact’ based on gene expression profiles may, in reality, be physically distant within the tissue, or conversely, adjacent cells might not exhibit complementary ligand-receptor expression in transcriptomic data. To address this, spatial transcriptomics techniques have been developed to preserve the tissue architecture while capturing gene expression. Methods such as Slide-seq [54], 10x Visium [55], high-definition spatial transcriptomics (HDST) [56], and dendrimeric DNA coordinate barcoding design for spatial RNA sequencing (Decoder-seq) [57] enable spatially resolved gene expression profiling by either mapping sequencing reads to known positions on a slide or performing *in situ* RNA sequencing within the tissue. The outcome is a comprehensive map of gene expression across a tissue section (Fig. 5b). From such data, researchers can identify neighborhoods of cells and analyze the local enrichment of ligand and receptor transcripts. For instance, if genes associated with a particular signaling pathway are co-localized in adjacent spots, such as a cluster of cells expressing a chemokine next to another expressing its receptor, it strongly suggests actual cell-cell communication in that locale. Indeed, spatial transcriptomics has unveiled structured ‘microenvironments’ in tumors, such as immune-rich peripheries where T cells express activation markers adjacent to tumor cells expressing checkpoint ligands, contrasting with hypoxic cores dominated by different interactions. Integrating spatial data refines the multitude of potential interactions to those most likely relevant *in vivo*, enhancing confidence in predictions of cell-cell crosstalk. Adding further layers, spatial proteomics and multiplex RNA-protein imaging can directly validate the presence and co-localization of protein counterparts of transcripts on interacting cells. Techniques like MERFISH (multiplexed error-robust fluorescence in situ hybridization) [58] and seqFISH (sequential fluorescence in situ hybridization) [59] can visualize hundreds of RNA species *in situ*, often alongside protein markers, combining the breadth of omics with single-cell spatial resolution. These comprehensive maps facilitate the identification of multicellular structures such as tertiary lymphoid structures in tumors or stem cell niches in regenerative tissues, and crucially, the signaling molecules that sustain them.

Beyond static mapping, single-cell approaches can also capture dynamic responses to interactions. For example, co-culture experiments followed by single-cell sequencing, sometimes referred to as ‘paired-tag’ or physically interacting cells sequencing (PIC-seq), can profile two interacting cell types together [60]. In one study, malignant cells were co-cultured with macrophages; both cell types were then sorted as doublets and subjected to single-cell RNA-seq. This approach revealed how each cell’s transcriptome changed due to the interaction, identifying specific genes induced only upon contact between the two cells. Such designs help transition from correlation to causality, not only demonstrating that a macrophage could signal to a tumor cell, but also observing the actual gene programs activated in the tumor cell upon macrophage attachment, and vice versa. To further elucidate the temporal dynamics of cell-cell interactions, Zman-seq has been developed as a time-resolved single-cell RNA sequencing technique [61]. By introducing fluorescent time stamps into circulating immune cells, Zman-seq enables the recording of transcriptomic changes over time as cells infiltrate tissues (Fig. 5c). For instance, in glioblastoma, Zman-seq revealed that within 24 hours of tumor infiltration, cytotoxic natural killer (NK) cells transitioned to a dysfunctional state regulated by TGFβ1 signaling. Additionally, infiltrating monocytes differentiated into immunosuppressive macrophages over 36 to 48 hours, characterized by the upregulation of suppressive myeloid checkpoints such as TREM2, IL18BP, and ARG1. This temporal resolution provides empirical measurements of differentiation trajectories, enhancing our understanding of immune adaptation and informing the development of more effective immunotherapies.

In summary, single-cell and spatial omics provide a systems-level perspective on cell-cell communication. They enumerate the parts list each cell carries, such as ligands, receptors, adhesion molecules, and use that to computationally reconstruct candidate interaction networks. When grounded in spatial context, these methods can pinpoint where interactions are likely occurring in tissues. The power of these approaches lies in scale, simultaneously covering all cell types and signals, which allows discovery of unexpected communication pathways and emergent network properties. The challenge, of course, is that these are largely predictive or correlative. Therefore, hypotheses from single-cell data often loop back to needing direct validation by the more targeted tools described earlier, like imaging, proximity labeling, etc. Increasingly, researchers are integrating these approaches: using scRNA-seq to generate an interaction hypothesis, then designing a proximity labeling experiment or a reporter cell line to test that hypothesis *in vivo*. Such interplay of unbiased discovery and mechanistic validation is accelerating our ability to decode complex multicellular systems.

Beyond these experimental tools, artificial intelligence based *in silico* prediction tools are important assistants in resolving cell-cell communication networks. Advanced computational methods such as AlphaFold have brought a new revolution to structural biology. The updated version AlphaFold 3 could model ligand-receptor interactions more precisely due to its diffusion-based architecture which is capable of predicting the joint structure of complexes including proteins, nucleic acids, small molecules, ions, and modified residues [62]. Using *in silico* methods to stimulate ligand-receptor interactions can help researchers to find out the potent interaction pairs from the omics experimental results and to understand the molecular mechanisms of cell-cell communications.

## TRANSLATIONAL APPLICATIONS OF MOLECULAR TOOLS

**Figure 6.**
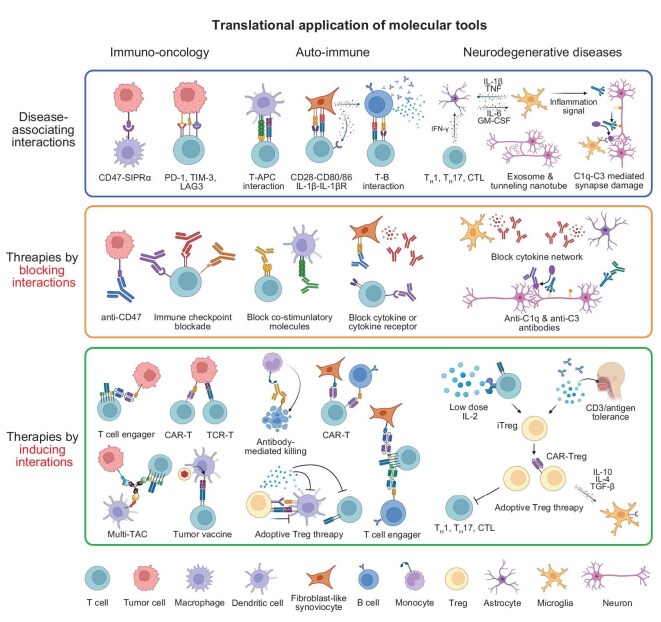
Examples of therapeutic strategies targeting cell-cell interactions in disease. In tumor immunology, cancer cells employ various interactions to evade immune surveillance: (1) expression of immune checkpoint molecules induces T cell exhaustion [63]; (2) interaction with macrophages via CD47 delivers a ‘don’t eat me’ signal [64]. Therapeutic strategies include: (1) antibody blockade of these interactions [63,64]; (2) enforced proximity between T cells and tumor cells using T cell engagers [65] or Multi-TACs [66] to promote cytotoxicity; (3) adoptive transfer of gene-edited T cells expressing CARs or tumor-specific TCRs [64]; and (4) tumor vaccination to enhance antigen presentation [67]. In autoimmune diseases, multiple interactions contribute to pathogenesis: (1) antigen-presenting cells (APCs), stromal cells, and B cells present self-antigens and activate autoreactive T cells via co-stimulatory receptors [68]; (2) autoreactive B cells secrete inflammatory cytokines and autoantibodies [68]. Therapeutic interventions include: (1) antibody blockade of co-stimulatory pathways [69]; (2) cytokine or cytokine receptor neutralization [70]; (3) depletion of B cells through antibody-dependent mechanisms [68]; (4) targeting stromal and B cells using T cell engagers (TCEs) or CAR-T cells [68], and suppressing APC or T cell activity via adoptive Treg transfer [71]. In neurodegenerative diseases, pathological interactions include: (1) proinflammatory crosstalk between microglia and astrocytes [72]; (2) activation of astrocytes by T helper 1 (TH1), T helper 17 (TH17) or cytotoxic T lymphocytes (CTLs) [73]; (3) complement-mediated synapse loss via C1q–C3 signaling from activated microglia; (4) propagation of pathogenic proteins through tunneling nanotubes and exosomes between neurons [74]. Potential therapies involve: (1) blockade the cytokine network [72] or the C1q–C3 signaling pathway by antibodies [75]; (2) induction of induced regulatory T cells (iTregs) via low-dose IL-2 or oral tolerance strategies [73]; and (3) adoptive transfer of Tregs or CAR-Tregs to mitigate CNS inflammation [73].

The molecular tools for decoding cell interactions are not only illuminating basic biology, but also being applied to address pressing medical problems. By mapping the cellular crosstalk underlying diseases, these approaches are uncovering new therapeutic targets and strategies (Fig. 6). In some cases, the tools themselves (e.g. engineered cells and proximity labels) have been adapted as treatments. Here we highlight how insights from cutting-edge cell-cell interaction technologies are translating from bench to bedside, in areas including immuno-oncology, regenerative medicine, and beyond.

### Immuno-oncology and immune therapies

Perhaps the most dramatic translation of cell-cell communication knowledge into therapy has occurred in cancer immunotherapy. Tumors are complex ecosystems where cancer cells interact with immune cells, stromal fibroblasts, blood vessels, and more collectively known as the tumor microenvironment (TME) [76]. Understanding these interactions has been pivotal in devising therapies that mobilize the immune system to fight cancer (Fig. 6). For example, the discovery that many tumors evade immunity by exploiting checkpoint receptors like PD-1 on T cells binding PD-L1 on tumor cells emerged from studies of T cell-cancer cell interactions. Blocking that interaction with antibodies—the basis of checkpoint inhibitor drugs—has led to durable remissions in multiple cancers. This landmark success of T cells interacting with tumor cells through PD-1/PD-L1 underscores how targeting specific cell-cell interactions can have game-changing clinical impact [77]. Using molecular tools can even help to predict the immune response during immune checkpoint blockade (ICB) therapy. Researchers have applied the imaging mass cytometry and logistic regression models to profile marker proteins in the TME from triple-negative breast cancer patients in PD-L1 therapy. They found that the best predictors of immunotherapy response are the proliferating CD8^+^TCF1^+^ T cells and MHC-II^+^ tumor cells, followed by B cells and granzyme B^+^ T cells. ICB responsive tumors contained abundant granzyme B^+^ T cells, whereas ICB resistant tumors were characterized by CD15^+^ tumor cells [78]. These results proved that *in situ* multicellular spatial organization and analysis methods could predict the therapeutic effect and assist precision immuno-oncology.

Likewise, engineering T cells with CARs to recognize a tumor antigen, such as CD19 on leukemia cells, forces a lethal immune cell-tumor cell interaction, resulting in tumor destruction [48]. CAR-T cell therapy, now approved for several blood cancers, is effectively a living drug that seeks out malignant cells and engages them in a synapse leading to their elimination. These breakthroughs were guided by decades of basic research mapping T cell interactions and are direct applications of modulating cell-cell communication for therapy.

Molecular profiling tools are now refining immunotherapy by identifying additional interactions in the TME that could be targeted. For instance, single-cell sequencing and spatial analyses of tumors have identified the dysfunctional states of exhausted T cells expressing multiple inhibitory receptors and immunosuppressive myeloid cells that physically associate with them. Recently, an advanced cellular interactome sequencing method (CINTER-seq) has been reported, which used a near-infrared light triggered photocatalytic proximity labeling chemistry to capture cellular interaction *in vivo* [79]. Notably, researchers found that tumor-interacting CD4^+^ T cells showed higher LAG3 expression than CD8^+^ T cells and LAG3 showed strong binding affinity for MHC-II. Thus, it could stabilize the interaction between CD4^+^ T cells and MHC-II^+^ tumor cells which is important in immunotherapy. Meanwhile, researchers also found that neutrophils were strongly activated through interactions with tumor cells to adopt a pro-tumor phenotype in an interaction-dependent manner.

New therapies are being developed to disrupt these suppressive interactions, such as antibodies blocking other checkpoint pairs like TIM-3 and LAG3, or drugs reprogramming macrophage-tumor cell crosstalk like blocking CSF1-CSF1R signaling that recruits tumor-associated macrophages. As another example, the interaction between CD47 on cancer cells and SIRPα on macrophages was identified as a key axis enabling tumors to avoid phagocytosis [64]; trials are now testing anti-CD47 agents to enhance macrophage attack on cancer cells, essentially removing a brake on a cell-cell interaction to stimulate immunity.

Proximity labeling, advanced imaging, and synthetic biology are rapidly influencing immunotherapy research and clinical translation. Methods such as LIPSTIC [30] are helping identify crucial cell subsets that interact during immune priming, providing actionable insights for vaccine design. Meanwhile, multiplex immunofluorescence is being increasingly employed in clinical pathology to quantify immune cell infiltration and tumor-immune contacts, offering predictive measures of patient responses to checkpoint inhibitors [28]. Complementing these diagnostic strategies, synthetic biology approaches—such as logic-gated CAR-T cells incorporating synNotch-like receptors—are enabling precise therapeutic interventions. By requiring recognition of dual tumor and stromal signals, these engineered T cells promise enhanced specificity, safety, and efficacy, highlighting the clinical potential of engineering targeted intercellular interactions [80].

### Regenerative medicine and tissue engineering

The regeneration of tissues and the engineering of transplantable organs depend on guiding cells to form proper interactions. Developmental biology has shown that stem cells require specific niches—microenvironments where support cells provide signals that maintain stemness or direct differentiation. Molecular tools are helping identify the components of these niches and enabling their recreation *ex vivo*. For example, single-cell analysis of bone marrow identified key interactions between hematopoietic stem cells and niche cells like mesenchymal stromal cells and endothelial cells, mediated by factors such as SCF-Kit ligand and CXCL12-CXCR4 [81]. By incorporating those signals or the niche cell types themselves into culture systems, researchers have dramatically improved the ability to grow stem cells or produce organoids that more closely mimic real organs. Organoid technology, growing miniature organs from stem cells, has benefitted from decoding the cell-cell interactions that occur during organ development. For instance, co-culturing developing brain organoids with microglia was found to be necessary to recapitulate proper neuronal maturation and circuit formation, since microglia provide critical trophic interactions with neurons [82]. Similarly, adding endothelial cells into organoid cultures often helps other cells self-organize by providing angiocrine signals and a scaffold for tissue architecture. These improvements stem from recognizing and reconstituting essential cell-cell communications in the dish.

In tissue engineering for therapy, such as building a skin graft or a cardiac patch, it’s not enough to have the right cell types present—they must also be arranged and signaled correctly. Tools like 3D bioprinting are being combined with knowledge of cell interactions to place cells in configurations that will encourage them to form junctions and functional units. For instance, printing alternating streams of hepatocytes and endothelial cells, which interact in the liver sinusoids, yields liver tissue constructs with higher functionality than random mixes, because the printed architecture promotes the native cell-cell contacts each cell type requires [83,84].

Bioorthogonal chemistry has even found translational opportunities here: the cell-adhesive click chemistry system mentioned earlier could, in principle, be used to enhance the engraftment of transplanted cells by covalently ‘locking’ them in contact with host tissue cells [85]. For example, one might modify therapeutic mesenchymal stem cells with a chemical handle and the target tissue with the complementary handle so that when delivered, the cells have a higher chance to lodge and integrate via bioorthogonal ligation. While not yet in clinical use, this strategy is being explored to improve cell therapies that suffer from poor retention at injury sites.

### Decoding cell-cell communications in disease to develop therapies

Beyond oncology and tissue engineering, a broad range of diseases are being re-examined through the lens of CCC, often revealing previously unappreciated therapeutic angles. In chronic inflammatory and autoimmune diseases, pathogenic interactions between immune cells and tissue resident cells drive damage. For instance, in rheumatoid arthritis, fibroblast-like synoviocytes in the joint aberrantly interact with T cells and macrophages to sustain inflammation (Fig. 6) [86]; single-cell studies identified specific signals like GM-CSF and IL-1 family cytokines in these cellular dialogs that can be targeted with existing drugs or new antibodies. Indeed, some therapies such as abatacept (CTLA-4-Ig) can be seen as targeting cell interactions: abatacept blocks CD80/CD86 on antigen-presenting cells from engaging CD28 on T cells, thus preventing a co-stimulatory interaction that propagates autoimmune T cell activation [87]. Similarly, in multiple sclerosis and other autoimmune diseases, the pathogenic role of certain T-B cell interactions has led to the use of B cell-depleting therapies and co-stimulation blockers that disrupt those cellular circuits.

In infectious diseases, considering cell interactions can guide interventions: HIV, for instance, spreads through direct cell contacts (virological synapses) between T cells [88]; strategies to interrupt these cell junctions or to protect the contacted cells are an active research area. During severe infections or sepsis, a dysregulated communication between immune cells, often an over-active ‘cytokine storm’, is what causes tissue damage. Profiling the immune cell interactome in such conditions has suggested that blocking specific interaction pathways can calm the dangerous crosstalk [89].

In neuroscience, neurodegenerative diseases are now appreciated as not just neuron-intrinsic disorders but as failures of neuron–glia interactions (Fig. 6). Microglia and astrocytes constantly survey and support neurons; faulty signaling between these cells is implicated in diseases ranging from Alzheimer’s disease to multiple sclerosis. For example, microglia may become overactive and strip away synapses after being stimulated by signals from stressed neurons or infiltrating T cells [90]. Investigations using single-cell RNA-seq of patient brains and mouse models have pinpointed interaction pathways, such as the complement cascade (C1q–C3), by which microglia recognize synapses for elimination in Alzheimer’s disease [75]. Experimental blockade of this microglia-neuron signaling pathway such as using antibodies against C1q or C3 has been shown to protect synapses in animal models, opening a possible therapeutic avenue [91]. Similarly, astrocytes interacting with neurons via inflammatory cytokines might drive neurodegeneration, and therapeutic efforts aim to modulate these neuron-glia communications. Beyond cellular interactions, even the spread of pathological protein aggregates in diseases like Parkinson’s may involve intercellular transfer mechanisms such as exosomes or tunneling nanotubes [74]. Thus, intervention strategies to block intercellular transmission of toxic proteins are being devised and tested [92].

**Figure 7.**
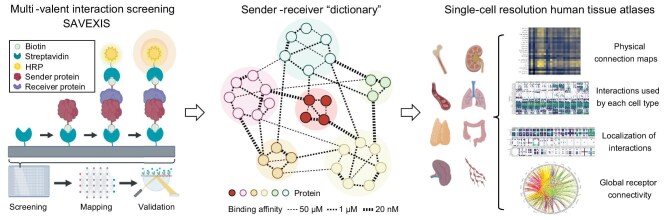
Wiring the intercellular interactome of the human immune system. SAVEXIS (scalable arrayed multi-valent extracellular interaction screen) is a high-throughput screening platform designed for efficient detection of protein binding interactions between recombinant extracellular domains. Subsequently, the interactome of immune receptors can be organized and the binding affinity can be further quantified. After integrating multicellular networks, physical connection maps, interactions used by each cell type, localization of interactions, and global receptor connectivity are available in the single-cell resolution human tissue atlases [89]. Copyright © 2022, Shilts *et al.* Published by Springer Nature.

These examples illustrate a common theme: by deciphering which cell-cell interactions go awry in disease, we can intervene more precisely. The growing toolkit for studying cell interactions accelerates this discovery process. Where once a pathogenic interaction might be hypothesized based on sparse evidence and artificial intelligence-based *in silico* prediction tools, techniques like multiplexed tissue imaging can directly show aberrant cell juxtapositions (e.g. autoimmune T cells in direct contact with pancreatic β cells in type I diabetes), and tools like proteomics or Interaction-Chip can identify the molecules pairing at those interfaces (Fig. 7) [31,32]. Already, this has led to novel therapeutic targets. One recent study mapping a ‘physical interactome’ of the human immune system uncovered new ligand-receptor pairs between immune cells that modulate responses, some of which are druggable and are being explored for immunodeficiency or cancer therapy (Fig. 7) [89].

It is worth noting that some molecular tools themselves can be adapted into therapies. For instance, viruses engineered for trans-synaptic tracing in neuroscience have inspired the design of viral vectors that deliver genes to connected neural circuits, potentially treating circuit-specific brain disorders [93,94]. Likewise, bispecific antibodies that physically link two cell types exemplify how chemical cell stapling concepts translate into clinical tools [94]. Blinatumomab, a bispecific T cell engager (BiTE) that tethers T cells to leukemia cells [65] is one of the most famous examples (Fig. 6). This principle extends further to next-generation platforms like multimodal targeting chimeras (Multi-TACs), which are modular agents assembled via triple orthogonal linkers (T-Linkers) [66] that co-engage multiple immune cells and tumor targets within the TME. For example, the EGFR-CD3-PD-L1 Multi-TAC not only bridges T cells and dendritic cells to enhance anti-tumor responses but also blocks PD-L1–mediated immunosuppression, addressing TME complexity in solid tumors (Fig. 6). These advances illustrate how programmable molecular engineering—from viral vectors to bispecifics to Multi-TACs—leverages intercellular interaction frameworks as direct therapeutic strategies.

In summary, the translation of cell-cell interaction science to medicine is well underway. As our ability to map these interactions improves, we can expect a growing pipeline of therapies aiming not just at cell-intrinsic targets but at the interfaces between cells, the crucial communication nodes of multicellular systems. By modulating these nodes, whether blocking harmful ‘conversations’ or promoting helpful ones, we tap into a level of control highly relevant to complex diseases. The following section will discuss the challenges that remain and new opportunities on the horizon for further integrating these tools and translating insights into practice.

## EMERGING OPPORTUNITIES AND CHALLENGES

Despite significant advances in studying multicellular communication, substantial challenges persist in fully capturing and understanding cell-cell interactions. Multicellular systems inherently possess remarkable complexity, with the human body comprising hundreds of distinct cell types engaging in dynamic, context-dependent contacts that continuously evolve across time, environmental changes, and disease conditions. Nevertheless, these challenges present significant opportunities for innovation, and numerous emerging technologies hold considerable promise for overcoming current limitations.

The current breakthrough in oncology and immunotherapy could also promote new biological research activities in other research fields. Neuroimmune interactions represent an especially compelling research frontier due to their critical roles in various physiological and pathological contexts. Despite growing interest, specialized tools tailored explicitly for studying neuroimmune communication remain limited, because the current approaches largely rely on neuroscience-derived methodologies such as chemogenetics and optogenetics which are not fully optimized to address the unique complexities of neuroimmune interactions [95]. The complex long-term cell-cell communication and interplay between nervous and immune systems are based on cells, secreted proteins, and metabolites which still calls for new techniques. Advanced molecular tools including chemical-omics probes, novel biosensors, and super-resolution real-time imaging methods can help to map and analyze the trajectories of the cells and messengers which can provide deeper insights into neuroimmune interactions. For instance, researchers recently applied high-resolution imaging, viral tracing, scRNA-seq and optogenetic tools to study neuroimmune circuits which could respond to lymph-borne inflammatory signals and identified some neuron subsets innervating lymph nodes, but secondary mediators and the other cell types participating in this circuit have not been discovered yet [96]. Therefore, application of these novel molecular tools will bring more significant biological discoveries as well as brand new questions to neuroimmunology. It may further potentially unlock new therapeutic strategies targeting neuroimmune pathways.

Current CCC results predominantly come from fresh samples. However, for decades, most of the clinical samples are preserved in formalin-fixed, paraffin-embedded (FFPE) blocks with poor biomolecule integrity especially for nucleotides. Application of advanced sequencing tools as well as spatial-omics and imaging methods to reconstruct CCC networks from clinical FFPE slices is another difficult but significant task, because it can help pathologists to dig more information from these rich yet underutilized treasures to assist disease diagnosis and treatment [97].

The advent of single-cell multi-omics technologies, which simultaneously integrate transcriptomic, proteomic, and spatial data from individual cells, holds significant potential for refining interaction inference [98]. Advances in single-cell proteomics may soon enable direct measurement of ligand-receptor interactions at the protein level, overcoming limitations associated with RNA-based inference [99]. It could enable real-time tagging of cell-cell interactions in living organisms [100]. Furthermore, high-throughput microfluidic co-culture platforms further facilitate systematic, parallel screening of cell-cell interactions, transforming complex multicellular studies into experimentally manageable workflows through automated imaging and sequencing.

Current methodologies still fall short of addressing these intricate dynamics comprehensively. A major hurdle in unraveling multicellular interactions is the complexity associated with data integration. Modern experimental approaches generate enormous datasets, ranging from high-resolution imaging data capturing millions of cell-cell interactions across entire organs, to single-cell multi-omics datasets profiling thousands of molecular features in tens of thousands of individual cells. Extracting meaningful biological insights from these vast and diverse datasets requires sophisticated computational approaches. Effective algorithms must seamlessly integrate spatial data with molecular profiles, accurately discerning when adjacent cells exhibit corresponding ligand-receptor signals and linked functional alterations. Recent advances in unsupervised machine learning methods have shown promise by clustering cells based not only on cell type but also on spatial proximity and predicted interaction partners [101]. Computational tools continue to evolve, aiming to statistically infer multicellular communication networks from complex single-cell data. However, distinguishing biologically meaningful interactions from background noise remains a considerable challenge, as inferred interactions frequently contain false positives or biologically irrelevant signals. Experimental validation remains critical yet laborious, demanding iterative cycles of computational predictions and experimental verification. To address these challenges practically, the development of standardized atlases mapping cell-cell interactions in healthy tissues can serve as valuable references against which disease states can be compared [60]. Initiatives such as the Human Cell Atlas are progressively incorporating cell interaction data, though these efforts remain at an early stage [102].

In parallel, experimental methodologies face specific technical limitations, further complicating comprehensive analyses. Imaging techniques, although providing exceptional spatial detail, typically exhibit low throughput and limited generalizability, hindering the study of rare or transient events across numerous individuals or fields of view. Single-cell sequencing, despite its powerful molecular resolution, often loses critical spatial context and struggles to reliably detect low-abundance signaling molecules, particularly ligands with low mRNA expression [103]. Proximity labeling methods, which capture molecular interactions at cell interfaces, usually necessitate artificial setups involving transgenic enzymes or exogenous probes, limiting their practicality in clinical or patient-derived settings. Moreover, the dynamic nature of cell interactions poses additional complications: conventional static snapshots frequently miss transient but essential interactions, complicating causal inference. While capturing dynamic molecular interactions in real time remains challenging, emerging methods such as Zman-seq—a time-resolved single-cell transcriptomics approach—have begun providing temporal resolution by introducing molecular ‘time stamps’ into circulating cells [61]. This innovative technology enables researchers to track gene expression dynamics longitudinally *in vivo*, enhancing understanding of cellular differentiation trajectories and potentially informing improved immunotherapeutic strategies.

Achieving robust *in vivo* applicability of these technologies constitutes another significant challenge. While numerous approaches demonstrate efficacy in cultured cells or animal models, translating these directly into human clinical samples is considerably more complex. Genetic tagging strategies, for instance, face substantial barriers in clinical contexts, as do systemic chemical probes, due to issues such as delivery efficiency, toxicity, and metabolic clearance. Consequently, there is growing demand for non-invasive, clinically compatible diagnostic tools capable of reliably detecting ongoing cell interactions. Emerging approaches include circulating biomarkers reflective of active cellular interactions—such as specific junction proteins or exosomes carrying interaction-specific molecular cargo [104]—as well as advanced molecular imaging techniques like positron emission tomography (PET) scans using radiolabeled ligands designed to bind selectively at interaction sites [105]. For example, molecular imaging could detect early interactions between metastatic cancer cells and bone stromal cells, providing critical insights into metastatic progression.

In summary, new technologies for studying multicellular communication are enabling new biological research activities. Although decoding the complexities of multicellular systems continues to present significant challenges—particularly regarding data integration, technical limitations, and *in vivo* applicability—ongoing innovation in experimental and computational tools steadily addresses these barriers [106]. By directly confronting these hurdles, researchers are transforming limitations into opportunities, developing comprehensive methodologies that encompass multiple communication modalities, enhancing clinical compatibility, and establishing robust predictive models for multicellular behaviors. Collectively, these advances set the stage for increasingly precise and effective applications of our expanding knowledge of cell-cell interactions.

## TOWARD A FRAMEWORK FOR PRECISION MEDICINE IN MULTICELLULAR SYSTEMS

**Figure 8.**
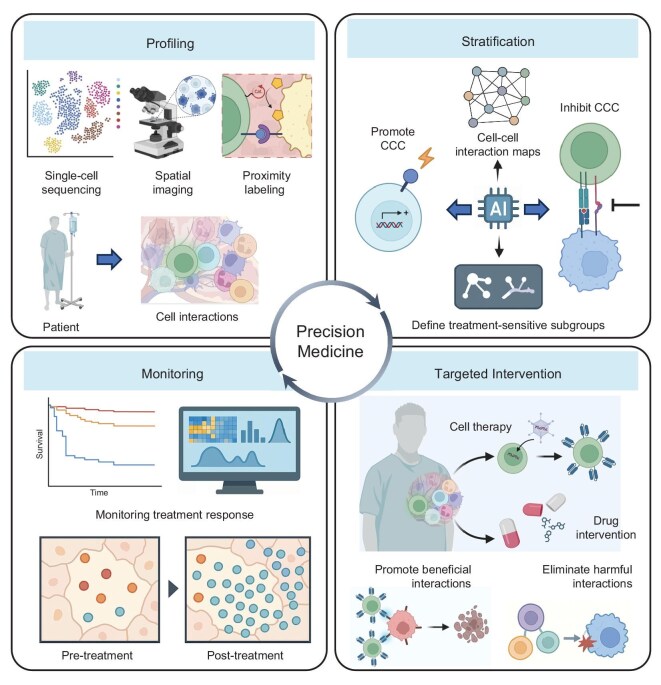
Precision medicine framework based on multicellular interactions. Profiling: single-cell sequencing, spatial imaging, proximity labeling, and other spatial methods define cell-cell communication networks of patients. Stratification: AI assists in identifying key interactions for subgroup classification which defines the treatment-sensitive subgroups. Targeted intervention: during drug or cell therapy, targeted interventions modulate pathogenic or reparative interactions, promoting beneficial interactions and eliminating harmful ones. Monitoring: post-treatment response monitoring evaluates cellular network reprogramming to guide adaptive therapy.

The ultimate promise of decoding multicellular communication is the ability to translate that knowledge into precision medicine—interventions tailored to the specific cellular interaction landscape of an individual’s disease [107]. Traditionally, precision medicine has focused on the genomic and molecular features within diseased cells, for example, targeting a cancer’s driver mutation with a specific inhibitor [108]. We now appreciate that an individual’s disease course and therapeutic response can also be profoundly influenced by the makeup and interactions of their cells. Two patients with the ‘same’ diagnosis may have very different cellular ecosystems—different compositions of immune cells, stroma, and signaling loops, and thus may respond differently to treatments. A framework that accounts for these multicellular features could greatly refine personalized therapy design (Fig. 8).

A precision medicine approach to multicellular systems would involve several steps: profiling, stratification, targeted intervention, and monitoring. First, the patient’s relevant tissue, like tumor and inflamed organ, would be profiled with high-dimensional tools to map its cellular composition and interaction network. This could include single-cell sequencing to identify cell types and their expressed ligands/receptors [109], spatial imaging to see how these cells are arranged and which are contacting each other [110], and possibly functional assays like organoid co-cultures to test how cells behave together [111]. The result would be a patient-specific cell-cell interaction atlas, effectively a blueprint of the cellular players and their communications driving that individual’s disease.

Next is stratification: analyzing that atlas to determine what the key pathways or cellular hubs are in this patient, and stratifying the patient into a subgroup that might benefit from certain therapies. For instance, in cancer, one patient’s tumor might be heavily infiltrated by macrophages forming an immunosuppressive interaction with tumor cells through high PD-L1 and IL-10 [112], while another’s tumor might instead be dominated by cancer–fibroblast interactions creating a protective niche (high TGF-β, dense collagen) [113]. The optimal therapy for the first patient might be an immune checkpoint inhibitor plus a macrophage-modulating agent, whereas for the second patient an anti-fibrotic or TGF-β blockade could be more effective. These decisions would be guided by comparative analysis against databases of prior patients: one would identify patterns. Already, studies in oncology are moving toward this direction, identifying ‘immune-high’ vs ‘immune-low’ tumors or categorizing them by which inhibitory ligands are most upregulated [114]. The framework extends similarly to other diseases: e.g. classify an autoimmune disease patient by whether their pathology is neutrophil-driven [115] vs T-cell-driven [116] based on cellular profiling, then choose therapy accordingly.

Crucially, this framework depends on having robust reference maps and validated biomarkers of cell interactions. As our molecular tools identify interaction signatures, such as a set of chemokines that indicate a feedback loop between two cell types, those can become biomarkers measured clinically. For example, a high plasma level of CXCL13 might indicate active T-B cell interactions in lymphoid tissues [117], suggesting a patient’s disease involves that axis and could benefit from disrupting it. Some interaction biomarkers might be tissue-based, requiring biopsy and advanced pathology analysis. The ongoing challenge is simplifying the high-dimensional data into a few key readouts that can guide decisions, effectively distilling the complexity into a clinically actionable form.

Once a target interaction or set of interactions is identified, the next step is targeted intervention. Therapies could be drugs that interfere with or boost specific cell-cell signals, or cell therapies that adjust the cellular makeup. Because multiple interactions often contribute to disease, combination therapies might be chosen to hit several nodes identified in the patient’s network. The framework envisions using the patient’s own interaction map to simulate or predict which combination is most likely to reset the network to a healthy state. For example, in a fibrotic lung disease, the map might show that epithelial cells, fibroblasts, and macrophages are in a pathological feedback loop: epithelial injury triggers macrophages, which activate fibroblasts, which in turn damage epithelia further [118]. A combination therapy might involve an anti-TGF-β plus a drug that promotes epithelial repair, rather than just a single agent. By targeting multiple interacting cell types simultaneously, something learned from cancer immunotherapy, where often two checkpoints are blocked at once or a checkpoint plus a vaccine is given—one can more effectively tip the balance of the whole system [119].

In some cases, the intervention might be to add or remove a cell population. For instance, if a certain regulatory cell type that normally keeps others in check is missing in a patient, one might infuse cells of that type to restore order. This concept is seen in ongoing trials where regulatory T cells are given to autoimmune patients, aiming to reinstate proper immune-suppressive interactions with overactive effector T cells [120]. Conversely, if a deleterious cell interaction involves a rogue cell population, such as pro-inflammatory T cell subset driving Crohn’s disease by interacting with gut macrophages [121], therapies might specifically deplete or reprogram those T cells to break the cycle.

Monitoring is the final aspect: after intervention, the same molecular tools can be used to check whether the targeted cell-cell interactions have indeed been altered as expected. This is a new kind of pharmacodynamics, not just measuring if a tumor shrinks or blood markers drop, but directly observing changes in the cellular crosstalk. If an immunotherapy is given, one could do a post-treatment biopsy and use spatial transcriptomics or multiplex imaging to see, for example, whether previously excluded T cells are now found in contact with tumor cells. Similarly, single-cell RNA-seq of peripheral blood might show that a previously overactive monocyte-T cell signaling gene module has diminished after an anti-cytokine therapy, confirming target engagement at the interaction level. In the precision medicine framework, this monitoring allows adaptive management—if an intended interaction change is not seen, one might switch strategies early.

Implementing this vision widely will require continued development. We will need faster, cost-effective assays for routine profiling, sophisticated computational decision-support systems, and clinical trials designed to test therapy selection based on cellular interaction features. Encouragingly, some elements are already falling into place. In oncology, the concept of the ‘immune phenotype’ of tumors is influencing trial designs [122]. In immunology, analyzing the complexity of immune infiltrates is guiding combination therapy choices for autoimmune diseases [123]. Pharmaceutical companies are increasingly including exploratory single-cell or spatial analyses in trials to identify responders vs non-responders based on cell interactions [124]. Moreover, as molecular tools become more miniaturized and automated, one can imagine a future where a patient’s biopsy is quickly subjected to a standardized panel of single-cell and spatial assays, even lab-on-a-chip devices that perform multiplexed imaging and sequencing in a day [124]. The results would be interpreted by a trained AI algorithm referencing a vast database of known interaction patterns and outcomes, yielding a report like: ‘Patient’s disease is driven by high macrophage-fibroblast crosstalk and T cell exclusion; recommend therapy combination X, which in similar profiles showed 80% success.’ While ambitious, this is a logical extension of current trends in precision oncology molecular boards, expanded to the cellular ecosystem level.

Precision medicine in multicellular systems also entails a shift in how we think of drug targets. It suggests we may increasingly target interfaces rather than single molecules, for example, a drug might be developed to sterically hinder the immunological synapse formation itself, not just block one receptor, or a therapy might aim to alter the physical properties of the tumor extracellular matrix to prevent certain cell contacts. It broadens the therapeutic design space to include modulating spatial and mechanical aspects of cell interaction, for instance, using a drug to promote blood vessel normalization in tumors, thereby allowing immune cell infiltration [125], i.e. enabling an interaction indirectly. In the long run, the knowledge gained from decoding multicellular interactions could enable preventive precision medicine: identifying individuals at risk of disease based on subtle interaction imbalances and intervening early. For example, if blood or tissue markers indicated that an otherwise healthy person has an emerging pattern of pro-inflammatory cell interactions [126], lifestyle or prophylactic measures could be advised to counteract that before it manifests as clinical disease.

In conclusion of this section, a framework for precision medicine that embraces the multicellular nature of disease is on the horizon. It will leverage the rich information provided by molecular interaction mapping tools to stratify patients and personalize therapies in a way that accounts for the cellular context, not just cell-intrinsic mutations. Achieving this will require tight integration of biotechnology, computational biology, and clinical research. Yet, as the tools described in this review continue to mature and penetrate translational research, the prospect of truly context-aware, interaction-targeted medicine is becoming increasingly tangible.

## CONCLUSION

Once considered an impossibly complex ‘black box,’ the multicellular ecosystem of tissues is rapidly yielding its secrets to an array of powerful molecular tools. Techniques spanning advanced imaging, bioorthogonal chemistry, genetic engineering, and single-cell omics are allowing us to observe and manipulate cell-cell communications with a level of detail and control unthinkable just a few years ago. These approaches have illuminated fundamental principles of how cells cooperate and compete—from the choreography of immune cells rallying together to fight infection, to the whispered growth signals exchanged in developing organs, to the insidious hijacking of communication channels by diseases like cancer [1]. What is emerging is a picture of biology that is fundamentally interactive and interconnected: phenotypes and pathologies are not the product of solitary cells acting alone, but of networks of cells engaging in molecular conversation.

The impact of this paradigm shift is twofold. First, on the scientific front, we now have the tools to map the cellular interactome of complex systems, effectively decoding the ‘language’ cells use to influence one another. Each tool adds a piece to the puzzle—imaging reveals who is talking; proximity labeling tells us in what molecular dialect; single-cell sequencing provides the transcripts of the conversation; synthetic circuits let us ask ‘what if’ by altering the dialog. By integrating these, we approach a holistic understanding of tissue function as an emergent property of intercellular networks. Achieving this understanding across different organs and organisms is a grand challenge for the years ahead, but one that seems within reach given the trajectory of technological innovation. Notably, as we chart these interaction landscapes, we often find recurring motifs—akin to common phrases in the language of cells, such as certain signaling loops, like Notch–Delta lateral inhibition or M1 macrophage–Th1 cell positive feedback [127,128], showing up in multiple contexts. Identifying these may lead to unifying principles or ‘rules of engagement’ that govern multicellular organization.

Second, on the translational front, we are witnessing a convergence of this knowledge with medical innovation, heralding a new era of interaction-centric therapies. The successes in immunotherapy and regenerative medicine underscore that intervening at the level of CCC can yield potent and sometimes curative results. As our ability to diagnose the state of cellular ecosystems improves, treatment of disease will become more nuanced and precise. The physician of the future may routinely consider diagnoses such as ‘excessive T-B cell collaboration in lymphoid tissue’ or ‘deficient supportive neuron–glia interactions,’ and treat accordingly, rather than relying solely on broad histological categories. In this sense, the review and mastery of multicellular interaction networks is not an academic exercise but a practical necessity to fully deliver on the promise of personalized medicine.

Of course, significant work remains to translate bench insights into bedside tools. Many of the methods discussed are still primarily research tools, not yet standard clinical assays. But the pace of progress is brisk: multiplex imaging is already moving into clinical pathology labs, liquid biopsy approaches are beginning to capture signals of CCC, like clusters of circulating tumor cells bound to platelets or immune cells as prognostic indicators, and clinical trials are increasingly mechanism-driven by insights into cellular cross-talk. Interdisciplinary collaboration will be key to overcoming remaining barriers: chemists, biologists, engineers, data scientists, and clinicians must continue to work together to refine these tools, validate them in patient samples, and ensure they are accessible and interpretable in clinical settings. Importantly, ethical frameworks will need to keep up, especially as we consider cell engineering therapies that could have ecosystem-level effects in the body.

In conclusion, the study of multicellular systems is being transformed by molecular tools that let us peer into the microscopic conversations that define life. Each new technology, whether a novel proximity probe or a new sequencing method, adds a layer of clarity to our view of how cells collectively build organs, fight infections, and in cases of misfortune, drive diseases. With detailed understanding comes the power of intervention: to silence a harmful interaction, to amplify a beneficial one, or to redirect a miscommunication. The narrative that emerges ‘from mechanisms to medicine’ is one of increasing coherence between what we learn at the bench and what we apply at the bedside. Decoding multicellular systems not only satisfies our deep scientific curiosity about how cooperation and coordination emerge in biology, but also provides us with arsenals to battle diseases that arise from failed CCC. We now stand at an exciting nexus where molecular details meet human health. Moving forward, embracing the full complexity of multicellular life will lead to simpler and elegant solutions that improve life.
